# Adolescent Self-Control and Individual Physical and Mental Health in Adulthood: A Chinese Study

**DOI:** 10.3389/fpsyg.2022.850192

**Published:** 2022-04-04

**Authors:** Fan Yang, Yao Jiang

**Affiliations:** ^1^Department of Labor and Social Security, School of Public Administration, Sichuan University, Chengdu, China; ^2^Department of Demography, Zhou Enlai School of Government, Nankai University, Tianjin, China

**Keywords:** health benefits, self-control, cognitive effect, continuous effect, China

## Abstract

Despite its association with individual health, few studies have focused on the relationship between adolescent self-control and individual physical and mental health in adulthood. We aimed, therefore, to explore the impact of adolescent self-control on individual physical and mental health in adulthood. We employed the dataset of China Labor-Force Dynamics Surveys [CLDS] (2016). 13,389 respondents with an average age of 45.621 years are consisted in this study. The respondents were asked to report their adolescent self-control through recall. The ordered probit and the ordinary least squares (OLS) models were employed to estimate the effects of adolescent self-control on physical and mental health in adulthood, respectively. We adopted the propensity score matching (PSM) method to address the self-selection bias of samples. The results indicated that adolescent self-control significantly affected individual physical health (coefficient = 0.0126, *p* < 0.01) and mental health (coefficient = −0.1067, *p* < 0.01) in adulthood. The mechanism analysis suggested that adolescent self-control partially promoted physical and mental health in adulthood by education and income instead of self-control in adulthood. In conclusion, paying attention to the cultivation of self-control before adulthood may be an effective way to improve individual physical and mental health in adulthood.

## Introduction

Health is an important human capital and the basis of other types of human capital, as well as a basic right and welfare of individuals. “Ensuring healthy lives and promote wellbeing for all at all ages” is one of the 17 sustainable development goals and targets in Transforming our World: The 2030 Agenda for Sustainable Development released by United Nations (2015). The influencing factors of individual health have always been the focus of academic attention, and relevant research has extended from the material level such as diet, physical exercise, and climate environment to the psychological and spiritual fields such as work and life stress (Hammer and Sauter, 2013; Mosadeghrad, 2013; Jiang et al., 2020; Yang et al., 2021).

The purpose of this paper is to explore how adolescent self-control (at the age of 14) affects individual physical and mental health in adulthood in an Eastern context. As a psychological concept rich in meaning, self-control generally refers to the individual’s capacity to override impulses, break habits, and avoid temptations (Hagger et al., 2019). There is much evidence in psychological and behavioral research that higher self-control is associated with better personal behavior such as better academic achievements (Duckworth et al., 2019; Li et al., 2021), more interpersonal trust and friendlier interpersonal relationships (Righetti and Finkenauer, 2011), and better environmental adaptation (Tangney et al., 2004). However, weaker self-control is associated with worse personal behavior such as overspending (Gathergood, 2012), procrastination (Kim et al., 2017; To et al., 2021), gambling (Arneklev et al., 1993), academic fraud (Reisig and Pratt, 2011), drug abuse (Baler and Volkow, 2006), corruption (Wolfe et al., 2016), sexually transmitted diseases (Bryan et al., 2001), worse interpersonal relationships (Shea et al., 2013), and criminal activities (Donner and Jennings, 2014). Regarding long-term effects, self-control and its development in adolescence can predict many behaviors and perceptions in adulthood, such as love, relationships, well-being, and work, which have been verified by some studies (Converse et al., 2018; Allemand et al., 2019).

Since self-control dilemmas are common in the health context, numerous studies have tried to explore the relationship between self-control and individual health behavior such as physical activity, healthy eating, alcohol consumption, and unhealthy snacking (Forestier et al., 2018; Hagger et al., 2019; Francis et al., 2021). Most of these studies focus on the short-term effect of self-control on health behavior. An intertemporal follow-up study using dataset from New Zealand shows that self-control in childhood can predict physical health, wealth, and public safety in adulthood. However, it is based on a Western context and only predicts individual physical health (Moffitt et al., 2011).

We suggest that adolescent self-control affects individual physical and mental health in adulthood through two effects, viz., a cognitive effect and a continuous effect. Regarding the first one, cognition refers to the states and process of individuals acquiring or applying knowledge, which, as a whole, include perception and judgment (Britannica, 2021). Cognition is the most basic psychological process of human beings (Britannica, 2021). The human brain receives information input from the outside world, which is processed by the brain, transformed into internal psychological activities, and then dominates human behavior (Britannica, 2021). Self-control contributes to cognitive ability (Tangney et al., 2004; Duckworth and Seligman, 2006; Zhou et al., 2010; Hofer et al., 2012; Duckworth and Carlson, 2013; Galla et al., 2014; Heckman et al., 2014; Voyer and Voyer, 2014; Blair and Raver, 2015; Duckworth et al., 2015, 2019; Bertrams et al., 2016). A study of secondary school students showed that a higher self-control capacity predicts lower anxiety-impaired cognition during math examinations (Bertrams et al., 2016). Students with higher self-control have better academic performance (Duckworth et al., 2019). In the process of learning, self-control helps to resist impulse and temptation, maintain the focus on setting goals earlier, and improve academic performance and cognitive ability (Duckworth et al., 2019). Among the multitude of factors affecting academic achievement, kindergarten teachers distinguish self-control as the most essential factor for success in school (Blair and Raver, 2015). Furthermore, an ever-increasing body of empirical evidence has shown that self-control has a pervasive influence on academic attainment, academic course grades, and standardized achievement test scores (Duckworth and Carlson, 2013). Students with high self-control perform better than students with low self-control in the above aspects at almost all levels of schooling (Tangney et al., 2004; Zhou et al., 2010; Hofer et al., 2012; Galla et al., 2014; Heckman et al., 2014; Blair and Raver, 2015). Similarly, the developmental advantage on self-control is conducive to explaining why the grades of female students outperform male students at school despite minimal differences in standardized achievement tests, intelligence, or academic motivation (Duckworth and Seligman, 2006; Voyer and Voyer, 2014; Duckworth et al., 2015).

Thus, self-control is conducive to enhancing cognitive ability and academic performance (Tangney et al., 2004; Duckworth and Seligman, 2006; Zhou et al., 2010; Hofer et al., 2012; Duckworth and Carlson, 2013; Galla et al., 2014; Heckman et al., 2014; Voyer and Voyer, 2014; Blair and Raver, 2015; Duckworth et al., 2015, 2019; Bertrams et al., 2016). Individuals with high self-control are more likely to receive a higher level of education than individuals with low self-control (Duckworth et al., 2019). Better academic performance also means stronger cognitive ability, including the cognitive ability of health knowledge. Health knowledge can guide individual health behavior in daily life, so as to maintain physical and mental health. A study has found that individuals with high levels of trait self-control eat more healthily because they have higher self-efficacy, more positive taste expectations, stronger intentions, and more plans, compared to those who have low self-control levels (Hankonen et al., 2014). Individuals with low self-control also are more likely to become addicted to drugs, which will damage their health (Baler and Volkow, 2006; Tang et al., 2015). In addition, self-control facilitates the enhancement of education attainments, and this, in turn, leads to a stronger economic ability, such as a higher income (Converse et al., 2014; Brody et al., 2020). Thus, a stronger economic ability may produce higher levels of investments in health, and, ultimately, individuals’ health can be better maintained (Beenackers et al., 2018).

Regarding the continuous effect, a certain cognitive style formed in adolescence may persist into adulthood (Vaske et al., 2012). Similarly, self-control includes stability (Turner and Piquero, 2002). The General Theory of Crime proposed by Gottfredson and Hirschi (1990) contends that low self-control interacts with opportunity to produce criminal and analogous behaviors. They argue that once established between the ages eight to ten, self-control remains relatively stable over one’s life course. Further, in a four-month test-retest among university students, Arneklev et al. (1998) found that the self-control levels of respondents were relatively stable. The study carried out by Turner and Piquero (2002) on offenders and non-offenders also offers mixed support for Gottfredson and Hirschi’s stability postulate on self-control. Similarly, a study of five-wave longitudinal data of Korean youths between the ages of 10 and 14 also partially supports Gottfredson and Hirschi’s arguments on the relative stability of self-control (Jo and Bouffard, 2014). The study of Coyne and Wright (2014) on a sample of 360 twins and 423 non-twins found that self-control is identifiable and stable across early life, increasingly influenced by genes, and thus is a critical focus for early intervention.

In conclusion, self-control is relatively stable. When an individual has low self-control in their early years, it can be predicted that the individual will also have low self-control in adulthood. Therefore, according to the previous analysis, adolescent self-control can affect individual health in adulthood through a continuous effect. Based on the above analysis, we propose that adolescent self-control is closely related to individuals’ health in adulthood.

Our possible contributions to public health policy and the management research field are as follows. First, the results of this paper may allow a better understanding of the impact of individuals’ early self-control at the age of 14 on their physical and mental health. The results may also enhance our understanding of the determinants of individual health in a rapidly developing emerging economy and the world’s largest developing country. It is anticipated that a better understanding of these issues can aid further research, and help government pinpoint better public health management strategies for the improvement of adolescent self-control, and the maintenance of individual physical and mental health, particularly in China but perhaps also in other societies. Second, in terms of research methods, we used the propensity score matching (PSM) method to build a counterfactual framework and solve the self-selection bias of samples, to ensure the robustness of the results. Third, employing a mechanism analysis, we clearly revealed the paths of adolescent self-control affecting adult physical and mental health as seen through education and income.

## Materials and Methods

### Data Sources

We employed the China Labor-Force Dynamics Surveys (CLDS). (2016) dataset to test the proposed hypotheses (Yang and Jiang, 2020). The data were collected by the Center for Social Science Survey at Sun Yat-sen University from July to September 2016. The major objective for collecting the data was to provide basic public data for social science research in China. The data cover education, work, migration, health, economic activities, and other interdisciplinary aspects, and include data from 29 provincial administrative units. The survey adopted multistage cluster, stratified, probability proportionate to size sampling. Therefore, the data were nationally representative, and high-quality. We cleaned the data by excluding the missing values, outliers, and other abnormal values. Finally, we obtained 13,389 valid samples in this analysis.

### Measures

#### Explained Variables

The explained variables in this study involve physical and mental health. For the variable of physical health, we used self-rated health to measure it (Yang et al., 2021). The respondents were asked, “How would you rate your current physical health status?” The answers were assigned 1–5 on a five-point Likert scale, from “very unhealthy” to “very healthy.”

For the variable of mental health, the Center for Epidemiological Studies Depression (CES-D) scale was adopted to measure respondents’ mental health status (Radloff, 1977). The CES-D scale has been verified to be valid for the assessment of depression and mental health status in a Chinese context (Tang et al., 2019; Yang et al., 2021). The total score of the CES-D scale ranges from 20 to 80, and the higher the CES-D score, the deeper the depression, and the worse the mental health status.

#### Explanatory Variable

The core explanatory variable in this study is respondents’ adolescent self-control. We used the adolescent self-control scale developed by the CLDS. The scale includes three items about the respondents’ self-control at the age of 14: “Even if I feel a little sick or have other reasons to stay at home, I still try to go to school (go to school),” “Even if it is a lesson I do not like, I still try my best to learn it (learn lesson),” and “Even if it takes a long time to finish my homework, I still try my best to do it (finish homework).” The respondents were asked to answer the above three questions by recalling the situation at the age of 14. The answers of the three items are assigned 1–4 on a four-point Likert scale, from “strongly disagree” to “strongly agree.” To obtain the value of adolescent self-control, the answers of the three items were aggregated. Therefore, the value of adolescent self-control ranges from 3 to 12.

The validity of the above three items regarding respondents’ adolescent self-control was tested using the Polychoric correlation matrix. The Polychoric correlation matrix reported that the minimum correlation coefficient in the matrix was 0.6522. This indicates that aggregating the three items has good validity for the measurement of adolescent self-control.

#### Mediating Variables

As mentioned, we argue that adolescent self-control impacts individual physical and mental health as seen through their education, income, and adulthood self-control. Therefore, the education, income, and adulthood self-control of the participants play mediation roles in the impact of adolescent self-control on health outcomes.

Education is a continuous variable measured by the schooling years of the participants. Income is also a continuous variable obtained by the total annual income of the participants in 2015.

Values of adulthood self-control were obtained from the adulthood self-control scale developed by the CLDS. The scale includes three items about the respondents: “Even if I feel a little uncomfortable or have other reasons to rest, I still try to accomplish what I should do every day (accomplish routine duties),” “Even if it is something I do not like but my duty requires me to do it, I still try my best to do it (do something dislike),” and “Even if a thing takes a long time to come to fruition, I still try my best to finish it (finish things taking long time).” The answers of the three items are assigned 1–4 on a four-point Likert scale, from “strongly disagree” to “strongly agree.” Thus, the total value of the adulthood self-control ranges from 3 to 12.

Likewise, we also tested the validity of the adulthood self-control scale by employing the Polychoric correlation matrix. The results of the Polychoric correlation matrix showed that the minimum correlation coefficient was 0.5986, which indicates that aggregating the three items has validity for the measurement of respondents’ adulthood self-control.

#### Other Control Variables

To obtain accurate conclusions, we controlled general factors affecting individual health incorporating gender (male = 1; female = 0), age, education, religion (with religion = 1; non-religion = 0), marriage (with marriage = 1; non-marriage = 0), logarithm of income in 2015, lifestyles (having habit of smoking / drinking / exercise = 1; otherwise = 0) (Smith, 1999; Zhang et al., 2018; Jiang et al., 2020; Yang and Jiang, 2020; Yang et al., 2021), and regional effects.

### Data Analysis

The key variables explained in this study are the respondents’ physical and mental health. Physical health is discrete and ordered data assigned 1–5 on a five-point Likert scale, and the ordered probit estimation could be used to test the influence of adolescent self-control on physical health. In the ordered probit model, we assumed that physical health y* was the unobserved variable (latent variable) and y* was expressed in the following equation:


(1) $$y^{*}=\beta_{0}+\sum_{\text{j}=1}^{\text{k}}\beta_{\text{j}}\,{\text{x}}_{\text{i}}+\mu_{\text{i}}$$

where x_i_ is the explanatory variable, individuals’ adolescent self-control. The μ_i_ was assumed to be normally distributed across observations. y* is unobserved, and it only can be observed when the performance of individuals’ physical health is “1”, “2”, “3”, “4”, or “5”. Thus, what can be observed is as follows:


(2) $$y_{i}=\left\{ \begin{array}{c} =1\;i\,f\;y^{*}\leq 1\\ =2\;1<y^{\text{*}}\leq \mu_{1}\\ =3\;\mu_{1}<y^{*}\leq \mu_{2}\\ \cdots \;\cdots \\ =\text{J}\;y^{*}\geq \mu_{J-2} \end{array}\right.$$

where μ_*1*_, μ_*2*_, and μ_*j–2*_ are unknown parameters to be estimated with β_j_.

The explained variable of mental health is continuous; thus, the ordinary least-squares (OLS) regression model can be employed to estimate the influence of adolescent self-control on individual mental health. The OLS regression model in this study can be written as follows:


(3) $$\mathrm{Mental}\,\mathrm{health}=\alpha_{0}+\delta \,x+\vartheta \,Z+\epsilon$$

where M*entalhealth* is the explained variable. x is the explanatory variable, individuals’ adolescent self-control, and Z is a set of control variables. α_*0*_ is the intercept. δ and ϑ are coefficients, and ϵ is error term.

In this study, it can be said that there was no mutual causality problem. An individual’s level of adolescent self-control is a predetermined variable. It can affect individual physical and mental health in adulthood, while it cannot be affected by the variables in the model. However, individual physical and mental health are not random, but are the outcomes of self-selectivity produced by systematic differences. Therefore, the self-selectivity is still a problem that cannot be neglected. We used the PSM method to build a counterfactual framework and understand the effect of adolescent self-control on individual’s physical and mental health (Rosenbaum and Rubin, 1983). The underlying logic of PSM is to balance out differences in observed characteristics by matching comparable control samples (adolescent self-control being lower than 8) for the treatment group (adolescent self-control being equal or greater than 8). In this study, we first estimated logistic regression models using a maximum likelihood to obtain the propensity score values. Second, based on the propensity scores, we performed matches on individuals whose adolescent self-control was equal or greater than 8 in the treated group with several different matching methods. Finally, we used the matched samples to estimate the average treatment effect on the treated (ATT), which can be expressed as follows:


$$\text{ATT}=\text{E}\left[\left(\mathrm{Physical}{\mathrm{health}}_{1\,i}-\mathrm{Physical}{\mathrm{health}}_{0\,i}\right)|{\text{AS}}_{\text{i}}=1\right]=\text{E}\left\{\text{E}\left[\left(\mathrm{Physical}{\mathrm{health}}_{1\,i}-\mathrm{Physical}{\mathrm{health}}_{0\,i}\right)|{\text{AS}}_{\text{i}}=1\right],p\left({\text{Z}}_{\text{i}}\right)\right\}$$

$$=\text{E}\{\text{E}\left[\mathrm{Physical}{\mathrm{health}}_{1\,i}|{\text{AS}}_{\text{i}}=1\right],$$

(4) $$p\left({\text{Z}}_{\text{i}}\right)-E\left[\mathrm{Physical}{\mathrm{health}}_{0\,i}|{\text{AS}}_{\text{i}}=0,p\left({\text{Z}}_{\text{i}}\right)\right]\left|{\text{AS}}_{\text{i}}=1\right\}$$

$$\text{ATT}=\text{E}\left[\left(\mathrm{Mental}{\mathrm{health}}_{1\,i}-\mathrm{Mental}{\mathrm{health}}_{0\,i}\right)|{\text{AS}}_{\text{i}}=1\right]=\text{E}\left\{\text{E}\left[\left(\mathrm{Mental}{\mathrm{health}}_{1\,i}-\mathrm{Mental}{\mathrm{health}}_{0\,i}\right)|{\text{AS}}_{\text{i}}=1\right],p\left({\text{Z}}_{\text{i}}\right)\right\}$$

$$=\text{E}\{\text{E}\left[\mathrm{Mental}{\mathrm{health}}_{1\,i}|{\text{AS}}_{\text{i}}=1\right],$$

(5) $$p\left({\text{Z}}_{\text{i}}\right)-E[\mathrm{Mental}{\mathrm{health}}_{0\,i}|{\text{AS}}_{\text{i}}=0,p\left({\text{Z}}_{\text{i}}\right)]|{\text{AS}}_{\text{i}}=1\}$$

where *Physicalhealth* and *Mentalhealth* are explained variables, viz., respondents’ physical and mental health in their adulthood; AS_i_ denotes a binary treatment variable, specifically, taking a value of 1 for the adolescent self-control being equal to or greater than 8; otherwise, AS_i_ = 0. p(Z_i_) represents the propensity scores, and Z_i_ is a set of covariates.

Furthermore, we employed the Bootstrapping method (200 times) to test the mediation roles of the education, income, and adulthood self-control of the participants in the impact of adolescent self-control on individual health outcomes in adulthood (Hayes and Scharkow, 2013).

## Results

### Descriptive Analysis

Table 1 reports the definitions of the variables and the results of the descriptive analysis (*N* = 13,389). Of the explained variables, the average value for respondents’ physical health was 3.669 (SD = 0.955) on a five-point Likert scale ranging from 1 to 5, and the mean value for mental health was 26.916 (SD = 8.595) on the CES-D scale ranging from 20 to 80. The explanatory variable, adolescent self-control, ranged from 3 to 12, with a mean value of 5.519 (SD = 2.997).

**TABLE 1:** Descriptive statistics (*N* = 13,389).

Variable	Mean	Standard deviation	Min	Max
**Explained variable**				
Physical health	3.669	0.955	1	5
Mental health	26.916	8.595	20	80
BMI	0.461	0.499	0	1
Physical pain	1.966	1.130	1	5
**Explanatory variable**				
Adolescent self-control	5.519	2.997	3	12
**Mediating variable**				
Adulthood self-control	8.690	1.501	3	12
Education	8.773	4.246	0	23
Logarithm of income	9.784	1.238	3.689	14.931
**Control variable**				
Gender	0.550	0.498	0	1
Age	45.621	12.728	18	96
Religion	0.123	0.329	0	1
Marriage	0.872	0.334	0	1
Smoking	0.331	0.471	0	1
Drinking	0.242	0.428	0	1
Exercise	0.288	0.453	0	1

Regarding the mediating variables, adulthood self-control had an average value of 8.690 (SD = 1.501), and ranged from 3 to 12. Education ranged from 0 (illiteracy) to 23 years (doctor) with an average amount of 8.773 (SD = 4.246) which closes to a level of junior high school. Respondents’ logarithm of income in 2015 had a mean value of 9.784 (SD = 1.238), with a minimum value of 3.689 and a maximum value of 14.931.

Regarding the control variables on the socio-demographic characteristics of respondents, out of the total respondents, 55% of the respondents were male. The age of the respondents ranged from 18 to 96 years, with a mean value of 45.621 (SD = 12.728). A total of 12.3% of the respondents were religious, and 87.2% were married. 33.1% of the respondents had the habit of smoking, 24.2% drank alcohol, and 28.8% exercised regularly.

### Benchmark Regression

The ordered probit and OLS models were employed as benchmark regressions to estimate the effects of adolescent self-control on individual physical and mental health in adulthood, respectively, and the results are shown in Table 2. The results presented in column (1) of Table 2 were estimated using an ordered probit model without controls, while the results in column (2) were estimated after adding all controls. It can be observed that the results in column (1) and (2) both indicate that adolescent self-control is significantly and positively (coefficient = 0.0334, *p* < 0.01; coefficient = 0.0126, *p* < 0.01) associated with respondents’ adulthood physical health. The respondents with higher adolescent self-control tended to have better physical health in adulthood stage than those with lower adolescent self-control.

**TABLE 2:** Effects of adolescent self-control on physical and mental health in adulthood.

Variable	Physical Health		Mental Health	
	(Ordered probit)		(OLS)	
	(1)	(2)	(3)	(4)
Adolescent self-control	0.0334***	0.0126***	−0.1710***	−0.1067***
	(0.0045)	(0.0046)	(0.0375)	(0.0389)
Gender		0.0679**		−1.5095***
		(0.0277)		(0.1861)
Age		−0.0217***		0.0114
		(0.0011)		(0.0101)
Education		0.0056		−0.0965***
		(0.0041)		(0.0361)
Religion		−0.0308		0.7385***
		(0.0434)		(0.2629)
Marriage		0.0282		−1.0701***
		(0.0312)		(0.2617)
Logarithm of income		0.1263***		−0.5860***
		(0.0108)		(0.0919)
Smoking		0.0320		0.0700
		(0.0267)		(0.2171)
Drinking		0.0588*		0.0543
		(0.0323)		(0.1764)
Exercise		0.0948***		−0.4382**
		(0.0276)		(0.1823)
Region	Control	Control	Control	Control
*N*	13,389			
Pseudo R^2^	0.0505	0.0838		
R^2^			0.0688	0.0912

Robust standard errors in parentheses; ***p < 0.01, **p < 0.05, *p < 0.1.

Column (3) and (4) of Table 2 present the effects of adolescent self-control on respondents’ adulthood mental health using OLS regression without controls and adding all controls, respectively. The results of column (3) and (4) both show that adolescent self-control correlates to respondents’ adulthood mental health significantly (coefficient = −0.1710, *p* < 0.01; coefficient = −0.1067, *p* < 0.01). This means that the higher adolescent self-control, the lower the score on the CES-D scale assessed in adulthood, viz., the better the respondents’ adulthood mental health status.

### Dealing With Self-Selection Bias

While the results in Table 2 confirm the positive effect of adolescent self-control on physical and mental health in adulthood, the problem of self-selectivity was still not solved. Therefore, in this part, we employed PSM estimation to address it. The premise of using PSM is that the dataset should pass the balance test to ensure that, after matching, there is no systematic difference between the two groups, except for the core explanatory variable. After conducting the balance test, the bias ratio of all variables after matching was lower than 10%.

Table 3 shows the effects of adolescent self-control on physical and mental health in adulthood by adopting four types matching methods: nearest-neighbor matching with caliper matching, radius matching, kernel matching, and local-linear regression matching. In the PSM analysis of the effects of adolescent self-control on physical health in adulthood, the values of the ATT in the different matching methods were significant and positive. In the PSM analysis of the effects of adolescent self-control on mental health in adulthood, the values of the ATT were also significant. The results indicate that, after eliminating observable systematic differences, adolescent self-control still has significant and positive impacts on the physical and mental health in adulthood.

**TABLE 3:** Propensity score matching estimation of the effects of adolescent self-control on physical health in adulthood.

Method	Nearest Neighbor	Radius	Kernel	Local Linear Regression
Physical health (ATT)	0.0539**	0.0421**	0.0457***	0.0481**
	(2.33)	(2.39)	(2.61)	(2.08)
Mental health (ATT)	−0.6995***	−0.6332***	−0.5771***	−0.5821**
	(−2.69)	(−3.22)	(−2.95)	(−2.24)
Control variables	Control	Control	Control	Control
Treated	4,921	4,921	4,921	4,921
Untreated	8,468	8,468	8,468	8,468

ATT means the average treatment effect on treatment. T-statistics are reported in parentheses. The element number of the nearest-neighbor matching with a caliper was 1, the radius was set to 0.01 in radius matching, and kernel matching and local-linear matching used default kernels and bandwidth. ***p < 0.01, **p < 0.05.

### Robustness Check

#### Sub-Group Regressions

According to gender, the full samples were categorized into two sub-groups: male and female. The results of the sub-group regressions are provided in Table 4. It can be observed that adolescent self-control significantly and positively affects both the male (coefficient = 0.0132, *p* < 0.05) and female (coefficient = 0.0125, *p* < 0.05) sub-groups’ physical health in adulthood. Further, estimated by employing OLS models, adolescent self-control is also significantly correlated to the male (coefficient = −0.0784, *p* < 0.1) and female (coefficient = −0.1460, *p* < 0.01) sub-groups’ mental health in adulthood. The results indicate that the conclusion that higher adolescent self-control is correlated with better physical and mental health in adulthood is valid across different gender sub-groups.

**TABLE 4:** Effects of adolescent self-control on physical and mental health in adulthood in different gender sub-groups.

Variable	Physical Health		Mental Health	
	(Ordered probit)		(OLS)	
	(1) Male	(2) Female	(3) Male	(4) Female
Adolescent self-control	0.0132**	0.0125**	−0.0784*	−0.1460***
	(0.0059)	(0.0059)	(0.0445)	(0.0482)
Control variables	Control	Control	Control	Control
Region	Control	Control	Control	Control
*N*	7,360	6,029	7,360	6,029
Pseudo R^2^	0.0851	0.0925		
R^2^			0.0834	0.1050

Robust standard errors in parentheses; ***p < 0.01, **p < 0.05, *p < 0.1.

#### Alternative Explained Variables

The method of alternative explained variable was also employed to test the robustness of the results. As mental health was measured by the widely acknowledged CES-D scale, we mainly replaced the explained variable of self-rated physical health with respondent’s body mass index (BMI) and frequency of physical pain in the last month in order to re-examine the estimated results. The normal BMI standard for Asians is from 18.5 to 23 (WHO Expert Consultation, 2004; Yang and Jiang, 2020). As such, if the respondent’s BMI was in the range of 18.5 to 23, we coded BMI as 1, otherwise, 0. Further, the alternative explained variable of physical pain was ordered discrete data assigned 1–5 on a five-point Likert scale, from “never” to “always.”

Accordingly, we employed the probit model to estimate the effect of adolescent self-control on BMI, and used the ordered probit model to obtain the influence of adolescent self-control on physical pain. The results of the probit and ordered probit regressions are provided in Table 5. The results show that adolescent self-control has significant influences on BMI (coefficient = 0.0103, *p* < 0.05) and physical pain (coefficient = −0.0093, *p* < 0.1). A higher level of adolescent self-control is correlated with a greater possibility of the BMI being close to normal and less frequent physical pain.

**TABLE 5:** Effects of adolescent self-control on body mass index and physical pain in adulthood.

Variable	(1) BMI (Probit)	(2) Physical Pain (Ordered probit)
Adolescent self-control	0.0103**	−0.0093*
	(0.0043)	(0.0049)
Control variables	Control	Control
Region	Control	Control
*N*	13,389	
Pseudo R^2^	0.0305	0.0515

BMI is the body mass index. Robust standard errors in parentheses; ** p < 0.05, * p < 0.1.

### Mechanism Analysis

Using the Bootstrapping method (200 times), we empirically tested the intermediary effects of education and income on the impacts of adolescent self-control on individual physical and mental health in adulthood. The mediation effect model estimation results are shown in Table 6. The results show that the indirect effects of adolescent self-control on individual physical and mental health in adulthood as seen through education and income are both significant, and the direct effects of adolescent self-control on physical and mental health in adulthood are also significant. Thus, it is indicated that adolescent self-control partially promotes adulthood physical and mental health by the mediation effects of education and income. However, the indirect effects of adolescent self-control on adulthood physical and mental health through self-control in adulthood are not significant, which means adolescent self-control does not affect individual physical and mental health through self-control in adulthood.

**TABLE 6:** Mediating effects of education, income, and adulthood self-control in effects of adolescent self-control on physical and mental health in adulthood.

Variable		(1) Physical Health	(2) Mental Health
Education	Indirect effect	0.0016***	−0.0227***
		(0.0005)	(0.0049)
	Direct effect	0.0068**	−0.0599**
		(0.0027)	(0.0250)
Logarithm of income	Indirect effect	0.0006**	−0.0033*
		(0.0003)	(0.0019)
	Direct effect	0.0068***	−0.0599**
		(0.0025)	(0.0266)
Adulthood self-control	Indirect effect	0.0001	0.0002
		(0.0002)	(0.0018)
	Direct effect	0.0067**	−0.0601**
		(0.0027)	(0.0251)

Standard errors in parentheses; ***p < 0.01, **p < 0.05, *p < 0.1.

## Discussion

The present study suggests that adolescent self-control has a significant impact on individual physical and mental health in adulthood. Specifically, we found that a higher self-control ability in adolescence is correlated with better self-rated physical health, a greater likelihood of an individual’s weight being in a reasonable range, less physical pain, and a lower CES-D score assessed in adulthood. Whether studying the influence of self-control on academic performance in adolescence (Bertrams et al., 2016; Duckworth et al., 2019) or the influence of trait self-control, implicit self-control, and lay beliefs in self-control beliefs on some health-related behaviors (Hagger et al., 2019), they all remain in the short term, namely, the current influence of self-control on individual behavior results. In contrast to previous studies, this paper obtained the data of individual self-control in the early stage of life, specifically, in adolescence at the age of 14 years, through the memory of the respondents. Thus, we were able to further explore the lasting impact of adolescent self-control on individuals in adulthood. Its significance lies in revealing the profound influence of adolescent self-control on individual growth and development in an in-depth, long-range view. In particular, we observed self-control ability in late adolescence (14 years old). At this age, self-control becomes more mature and stable compared with childhood and early adolescence (Guo, 2018), and is more effective in predicting the individual situation in adulthood. In terms of physical health, the results in this paper are consistent with the findings of Moffitt et al. (2011) in New Zealand. It indicates that the influence of early self-control on adult physical health has commonalities in developed and developing countries.

Paying attention to the relationship between adolescent self-control and mental health in adulthood is another distinctive feature of this paper. In contrast to previous studies that pay attention to the impact of self-control on individual health behaviors such as physical activity, healthy eating, alcohol consumption, and unhealthy snacking (Forestier et al., 2018; Hagger et al., 2019; Francis et al., 2021), this paper has further discussed the impact of adolescent self-control on individual mental health in adulthood. In China, with the development of economy, people’s material living standards have been greatly improved; however, there are an increasing number of problems in the individual’s spiritual world. People face a lot of mental pressure in the fierce competition for survival and development (Wang et al., 2015). We found that adolescent self-control is conducive to regulating individual mental stress in adulthood. This is of practical significance to guide parents, especially new parents, regarding how to pay attention to their children’s mental health from the perspective of adolescent self-control.

In addition, we found that adolescent self-control mainly affects individual physical and mental health in adulthood as reflected in education and income. This verifies the cognitive effect of adolescent self-control on health in adulthood. Further, we found that adolescent self-control does not affect health through self-control in adulthood. In other words, adolescent self-control has a greater impact on individual health than that in adulthood. Therefore, the intentional training of individual self-control by governments, non-governmental organizations, and families should start from an early stage of life, not only in adulthood. Public institutions, especially hospitals and community schools, should publicize and educate new parents in the cultivation and training of self-control when children are young.

This study has some limitations. First, due to the lack of panel data, this paper selected one cross-sectional data. Although the participants were asked to recall their self-control at the age of 14, there may be biases in the memory of the respondents. In future studies, a more precise relationship between adolescent self-control and adult health may be captured through follow-up and continuous observation. Second, self-control is a complex psychological concept. Although we used an effective scale to measure it, its accurate measurement can be further studied to ensure more accurate results. Third, we have not discussed how to cultivate adolescent self-control, which is an important direction for future research.

## Conclusion

Using a nationally representative big data, this paper discusses the impact of adolescent self-control on individual physical and mental health in adulthood in China. Our results show that adolescent self-control affects not only the physical health, but also the mental health of adults. The effects are universal across all genders. Therefore, paying attention to the cultivation of self-control before adulthood may be an effective way to improve individual physical and mental health, and how to improve self-control in adolescence needs further research.

## Data Availability Statement

The data used in this manuscript are from the China Labor-Force Dynamics Surveys (CLDS) by the Center for Social Science Survey at Sun Yat-sen University in Guangzhou, China. The data analyzed in this study are available from the corresponding author on reasonable request.

## Ethics Statement

As the data employed in this study come from public databases, ethics approval was not required. Informed consent was obtained from all participants involved in the survey.

## Author Contributions

FY proposed the idea of this manuscript and wrote the introduction, theoretical analysis, discussion, and conclusion. YJ developed the method, wrote the results section, and modified and edited the whole manuscript. Both authors have read and agreed to the published version of the manuscript.

## Author Disclaimer

The opinions in this manuscript are the authors’ alone.

## Conflict of Interest

The authors declare that the research was conducted in the absence of any commercial or financial relationships that could be construed as a potential conflict of interest.

## Publisher’s Note

All claims expressed in this article are solely those of the authors and do not necessarily represent those of their affiliated organizations, or those of the publisher, the editors and the reviewers. Any product that may be evaluated in this article, or claim that may be made by its manufacturer, is not guaranteed or endorsed by the publisher.
